# Guyton perspective in managing peripartum cardiomyopathy patient with pulmonary edema: a case report

**DOI:** 10.1186/s13256-024-04398-2

**Published:** 2024-02-12

**Authors:** Ruth Evlin Margaretha, Yohanes W. H. George, Jefferson Hidayat

**Affiliations:** 1 Department of Anesthesiology and Intensive Care, Faculty of Medicine, Universitas Indonesia-Cipto Mangunkusumo General Hospital, Jakarta, Indonesia; 2 Semen Padang Hospital, Jl. Raya By Pass Km. 7, Kecamatan Pauh, West Sumatera Indonesia

**Keywords:** Peripartum cardiomyopathy, Furosemide, Negative cumulative fluid balance, Oxygenation improvement, Case report

## Abstract

**Background:**

Peripartum cardiomyopathy (PPCM) is a potentially life-threatening pregnancy-related condition characterized by left ventricular dysfunction and heart failure, typically occurring in the peripartum period. Individuals with a history of preeclampsia and hypertension are particularly prone to developing PPCM. Recent research suggests that the condition may be triggered by vascular dysfunction influenced by maternal hormones in the late stages of gestation. The onset of left heart failure results in decreased cardiac output, leading to insufficient perfusion, which in turn, contributes to pulmonary edema and exacerbates tissue hypoxia. This cardiovascular response activates the neurohumoral system, causing peripheral vasoconstriction and elevating both mean capillary filling pressure (MCFP) and central venous pressure (CVP). Early administration of furosemide reduces volume overload due to negative cumulative fluid balance gaining and vasodilation, which increases the velocity of intravascular refilling and causes interstitial edema to resolve. This will decrease interstitial fluid pressure, resulting in decreased mechanical compression to systemic capillary and systemic vein pressure, thus decreasing MCFP and CVP subsequently. Reduced CVP also contributes to increased venous return by decreasing the gradient pressure between MCFP and CVP, resulting in increased cardiac output (CO) and improved tissue oxygenation.

**Case:**

A 33-year-old Asian woman, para 3 at full term pregnancy, admitted to the intensive care unit (ICU) after c-section and tubectomy due to shortness of breath and palpitation. Based on history taking, physical examination and echocardiography the patient fulfilled the criteria of PPCM which was also complicated by pulmonary edema. Despite impending respiratory failure, the patient rejected intubation and continuous positive airway pressure (CPAP), and was given oxygen supplementation through nasal cannula. Furosemide was given rapidly continued by maintenance dose and CVP was monitored. Antihypertensive drug, anticoagulants, and bromocriptine were also administered. After achieving negative cumulative fluid balance the patient’s symptoms resolved and was discharged one week later.

**Conclusion:**

There is a correlation between negative cumulative fluid balance and reduced central venous pressure after early furosemide therapy. Suspicion for PPCM should not be lowered in the presence of preeclampsia, it could delay appropriate treatment and increase the mortality.

## Introduction

PPCM, a rare manifestation of pregnancy-related myocardial disease, is characterized by specific criteria: heart failure occurring in the last month of pregnancy or in the 5 months after delivery; no determinable cause of cardiac failure; absence of recognizable heart disease prior to the last month of pregnancy; echocardiographic findings of left ventricular dysfunction: left ventricular end-diastolic dimension > 2.7 cm/m^2^, M-mode fractional shortening ≤ 30%, left ventricular ejection fraction ≤ 0.45, [1–3] The critical timeframe for assessment spans from one month before delivery to five months postpartum. This period is crucial for ruling out pre-existing causes of cardiomyopathy that could be aggravated by pregnancy. PPCM incidence differed among all the reports from various countries. Incidence of PPCM range from ~ 1 in 1000 to 4000 live births in United States [4], whereas a 20-year study in Europe declares that the incidence is 1 in 4950 deliveries [5]. PPCM incidence differed among reports from various countries. A review in 2019 showed the highest incidence was in Nigeria (1 in 102 deliveries) and the lowest was in Japan (1 in 15,533) [6]. Risk factors associated with the development of PPCM include multiparity, advanced maternal age, multiple pregnancies, pre-eclampsia, gestational and pre-existing hypertension, and African–American ethnicity [7, 8]. Typically, affected women exhibit signs and symptoms of heart failure, such as orthopnea and paroxysmal nocturnal dyspnea. These manifestations can be mistaken for normal pregnancy symptoms, especially in late pregnancy, potentially leading to a missed or delayed diagnosis with serious consequences, including mortality rates ranging from 25 to 50% [4, 8]. More recently, it has been suggested that genetic factors may contribute to many cases of PPCM [9, 10].

## Case presentation

**Fig. 1:**
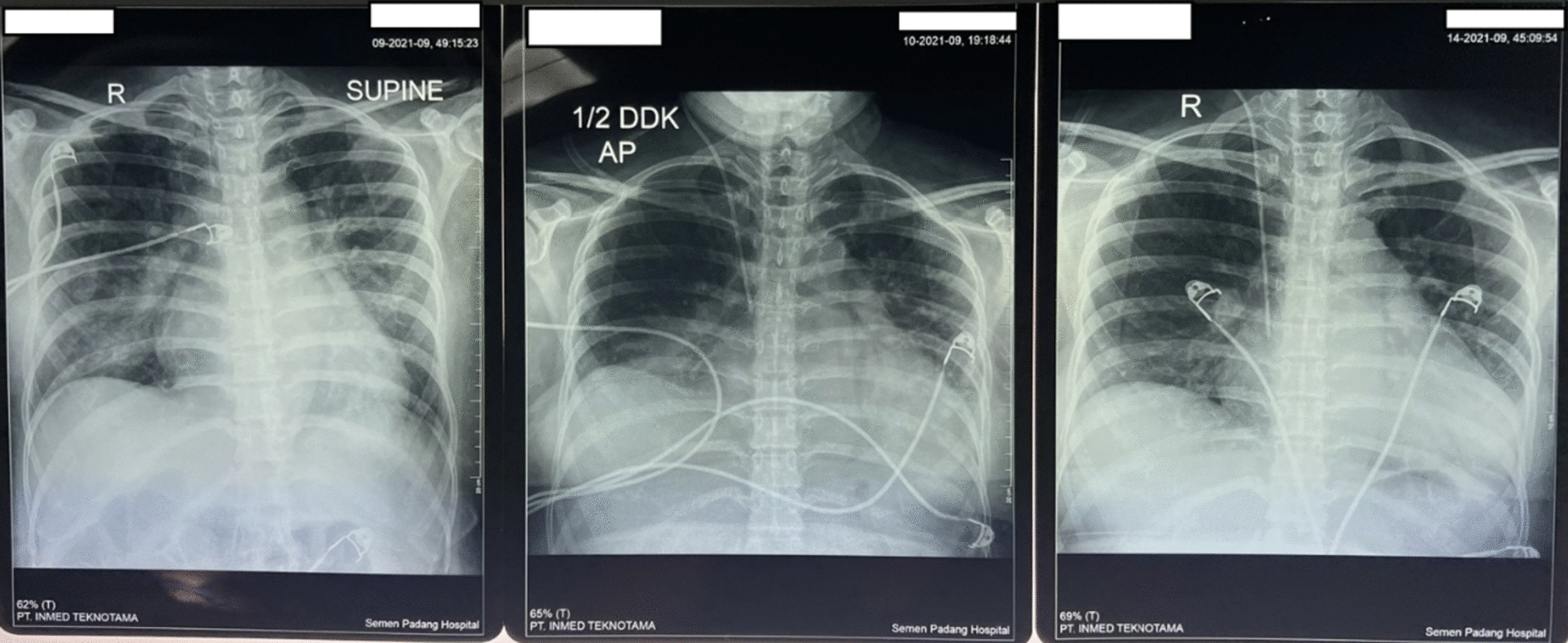
Chest X-ray examination (left: post operative day 0, middle: post operative day 1, right: post operative day 6)

**Fig. 2:**
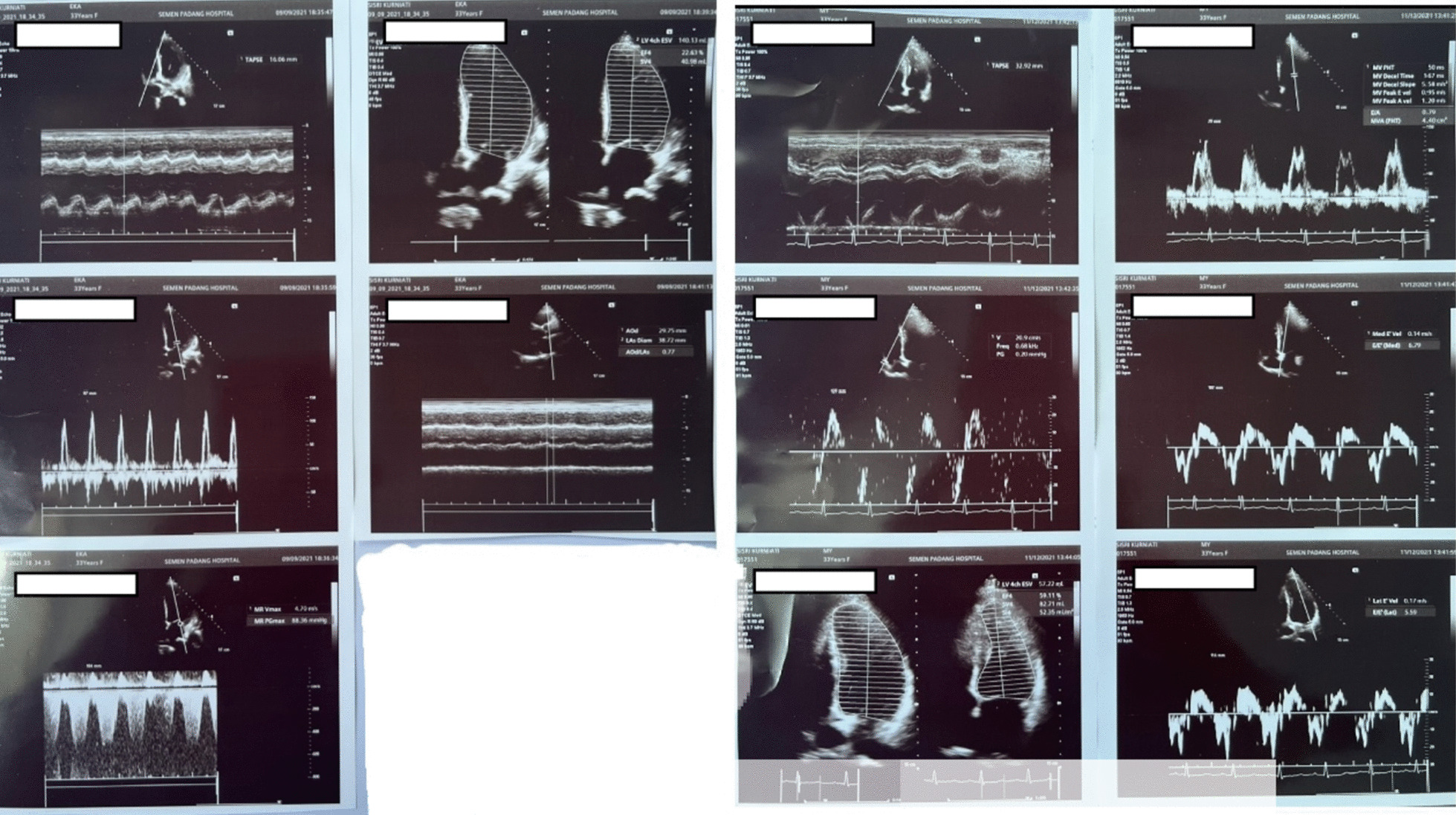
Echocardiography examination (left: post-operative day 0, right: 6 months later)

A 33-year-old Asian woman, in her third aterm pregnancy, ASA 2 with severe preeclampsia, was admitted to the ICU after caesarian section and tubectomy surgery due to shortness of breath and chest discomfort. The patient has a past medical history of severe preeclampsia in two previous pregnancies but no history of PPCM nor history of PPCM in her family, the patient also denied any history of congenital heart disease and history of heart failure. Her vital signs on ICU arrival were: blood pressure 136/92 mmHg, heart rate 150 beats/min, respiratory rate 38–40 breaths/min, temperature 38 ºC, saturation 95% with simple mask oxygen flow 6 L/min. She was awake and alert and was in a diaphoretic and tachypneic state. Lung examination revealed wet crackles throughout both lung fields. Her abdomen was diffusely tender. She had pretibial pitting edema. The neurologic examination yielded no abnormalities. Laboratory investigations at admission revealed white blood cell count was 20.300/mm^3^, hemoglobin and hematocrit were 13.6 g/dL, and 41% subsequently. Arterial blood gas analysis values on oxygen 6 L/min results were: pH 7.43; PaCO_2_ 27 mmHg; PaO_2_ 122 mmHg; PaO_2_/FiO_2_ ratio of 244; HC${\text{O}}_{3}^{ - }$ 17.7 mmol; BE − 3 mmol/L; SaO_2_ 98%. PT and aPTT were within normal limits, but the D Dimer value was increasing by 1999 ng/mL. Serum Na^+^ was 137 mmol/L; K^+^ 3.5 mmol/L; calcium total 9.9 mg/dL; ureum 25 mg/dL; creatinine 0.4 mg/dL; SGOT and SGPT within normal limits but her bilirubin values were increased (total 3 mg/dL; direct 2.5 mg/dL, indirect 0.5 mg/dL) and lactate 3.3 mmol/L. The electrocardiogram (ECG) showed sinus tachycardia with nonspecific ST-T wave changes. The chest X-ray examination showed cardiomegaly with increased interstitial markings (Fig. 1). Echocardiography revealed all chamber dilation with marked global hypokinesis, decreased systolic function of left and right ventricles, mild–moderate mitral regurgitation, with an ejection fraction of 22% (Fig. 2).

**Fig. 3:**
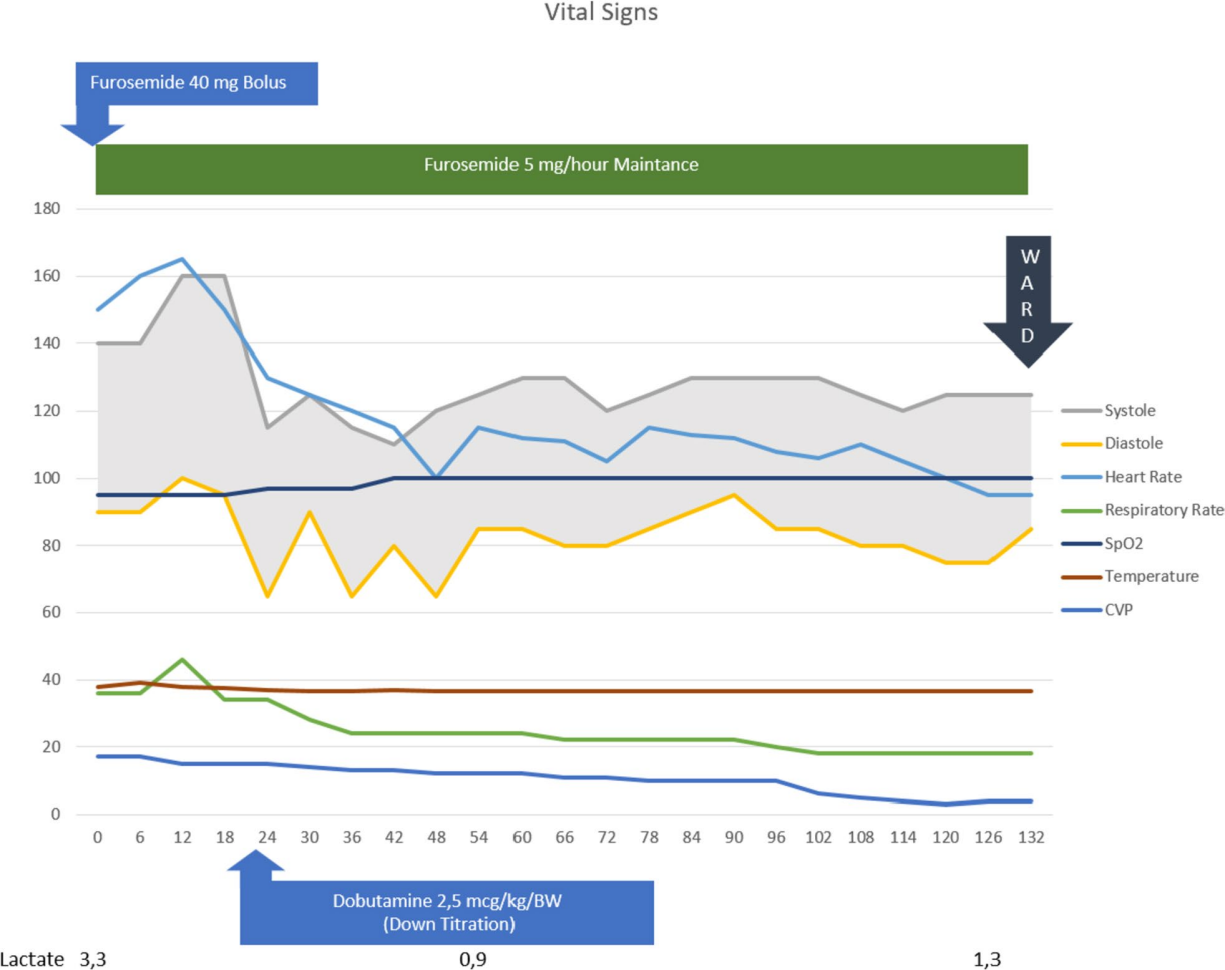
Timeline graphic of vital signs, CVP, lactate, furosemide and dobutamine dose consumption

From the history taking, physical examination and all the diagnostic examinations, the patient was diagnosed with PPCM and pulmonary edema. The patient refused intubation and CPAP despite having a respiratory failure (her respiratory rate 38–40x/min which would increase oxygen demand consumption and her PaO_2_/FiO_2_ ratio was 244). CVP was inserted and the initial value was 17 mmHg. Oxygen was given 6 liter per minute via simple mask and pulmonary edema was treated with Furosemide bolus 40 mg i.v. then continued with maintenance 5 mg/h with fluid balance target − 500 mL until − 1000 mL per day, Ramipril 2.5 mg every 24 h, Enoxaparine sodium 6000 iu (0.6 mL) every 24 h, Bromocriptine 2.5 mg every 12 h. Vital signs, CVP, and lactate was monitored strictly (Figure 3).

**Fig. 4:**
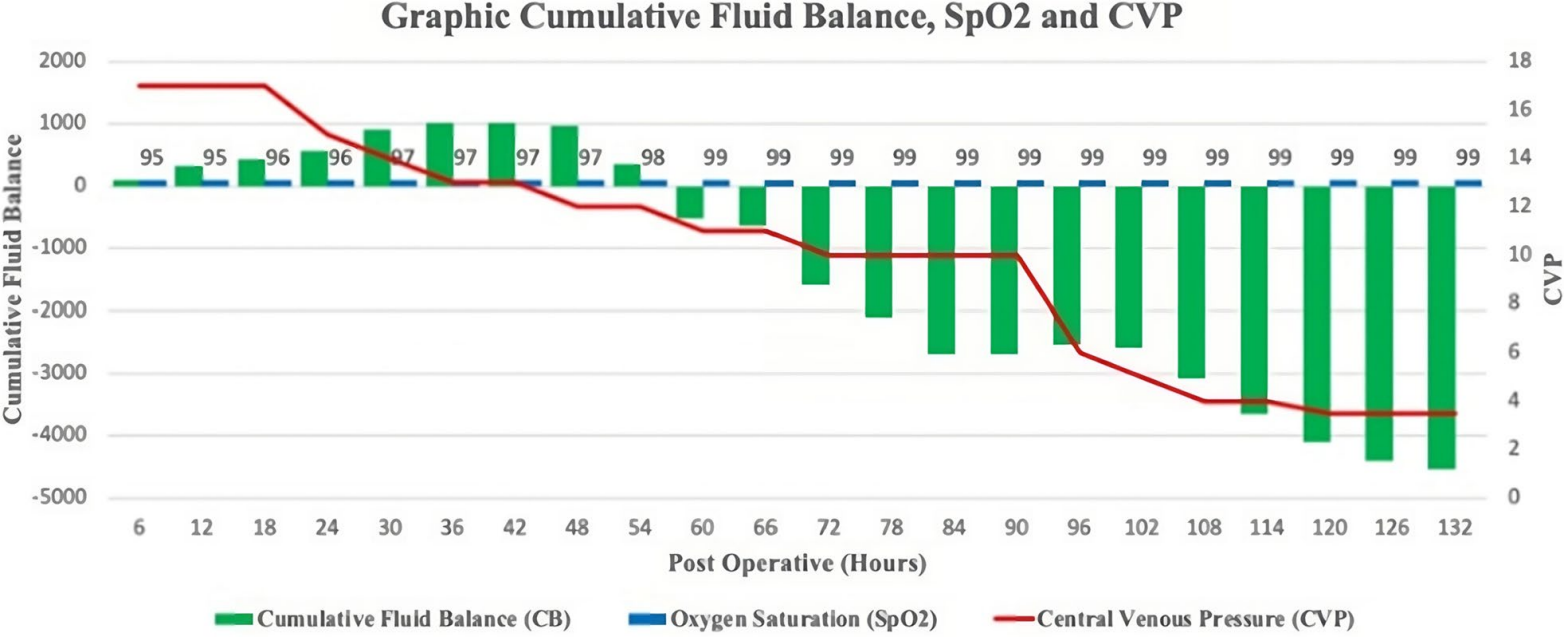
Graphic cumulative fluid balance, SpO_2_, and CVP

Fatigue and dyspnea were greatly relieved after gaining negative cumulative fluid balance (− 4540 mL/5 days) by Furosemide administration and CVP was decreased from + 17 mmHg to 3.5 mmHg (Fig. 4). She was discharged from the hospital one week later. Ramipril 2.5 mg and Bisoprolol 2.5 mg were instructed to be taken regularly at home. The patient’s adherence to medication was good and the patient did not experience any side effects from the medication.

The patient was satisfied with the treatment as her symptoms reduced two weeks after discharge and she had no symptoms 3 months after discharge. Home medications (Ramipril and Bisoprolol) were discontinued after three-months follow up. Six months after discharge, patient showed stable cardiomyopathy and well-controlled hypertension, and a repeat echocardiography at the same point showed an improved ejection fraction from 22 to 60%.

## Discussion

PPCM is characterized by dysfunction in the left ventricle and the development of heart failure [2, 3, 7]. In our case, the diagnosis criteria for PPCM were fully met. The left ventricle loses its normal contractile ability, leading to a compromised pumping function that hinders the heart's capacity to supply adequate blood flow and sustain sufficient oxygen delivery to meet the tissue's demand. This is primarily due to a decrease in cardiac output. Heart rate and stroke volume determine cardiac output, whereas the preload, contractility, and afterload determine the stroke volume [11–13]. In cases of low cardiac output, adjustments are made either in heart rate or stroke volume to ensure proper perfusion. If maintaining stroke volume becomes challenging, an increase in heart rate occurs to sustain cardiac output. In the observed patient, elevated sympathetic activity, indicated by vital signs, stimulated this response. Simultaneously, the cardiovascular system reacts to inadequate perfusion by activating the renin-angiotensin system, aiming to boost preload through salt and water retention, intensify vasoconstriction (thereby increasing afterload), and enhance cardiac contractility [11–13]. While this response is initially effective, prolonged activation leads to the loss of myocytes and adverse changes in surviving myocytes and the extracellular matrix. The myocardium, continuously exposed to stress, undergoes remodelling and dilation as a reaction to the insult. Remodelling further contributes to cardiac decompensation and gives rise to complications such as mitral regurgitation, caused by stretching of the valvular annulus. This complication was also evident in the echocardiography of the patient [11–13].

**Fig. 5:**
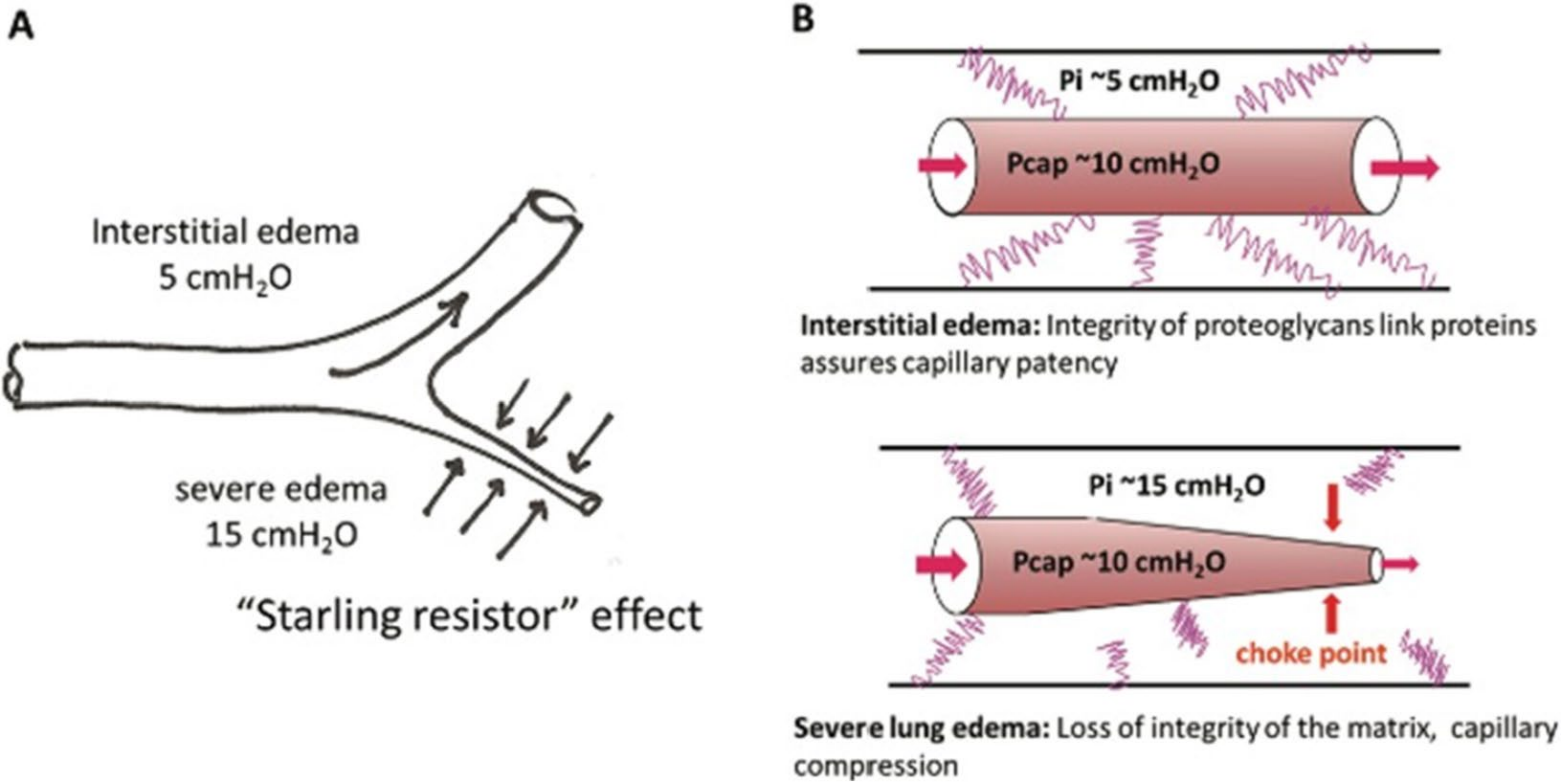
**A** Lung capillary squeeze in relation to interstitial fluid pressure (“Starling Resistor” effect). **B** Role of proteoglycans (in pink) fragmentation in favoring the squeeze of microvessels in severe edema

According to the heart–lung interaction theory, pulmonary edema is a consequence of elevated end-diastolic pressure. As the pressure rises, pulmonary capillaries are recruited to accommodate the additional volume, increasing their capacitance. As the pressure continues to rise, volume is redirected from the alveoli to the interstitium. At this stage, fluid formation takes place in the interlobular septae and the perihilar region due to the influence of pressure gradients [14, 15]. The development of interstitial edema leads to a compression of lung capillaries by the rising interstitial fluid pressure, a phenomenon known as the "Starling Resistor" effect. This compression narrows the diameter of lung capillaries, resulting in impaired blood flow (Fig. 5A) [14]. The reduction in the vascular bed causes an elevation in pulmonary vascular resistance, leading to an increase in pulmonary artery pressure. The magnitude of this pressure increase reflects the severity and extent of the edema (Fig. 5B).

According to Guyton's approach, CO is influenced by the interplay between cardiac function and systemic function venous return (VR). VR relies on the pressure difference between the mean systemic filling pressure (MCFP) and the right atrium pressure (RAP). Elevated RAP acts as a hindrance to VR. Clinical measurement of RAP is reflected in CVP. The disparity between MCFP and CVP establishes both VR and CO. In pulmonary edema, high CVP leads to an increased MCFP, aiming to sustain a constant pressure gradient for the maintenance of CO and tissue perfusion [16].

The treatment for PPCM aligns with strategies employed for other forms of congestive heart failure, involving measures such as fluid and salt restriction, beta blockers, and diuretics. However, angiotensin-converting enzyme inhibitors and angiotensin-receptor blockers are contraindicated during pregnancy [4, 8, 9]. Hydralazine, which reduces afterload, can be utilized in pregnancy. In this case, Ramipril was administered for anti-remodeling, considering that the patient had already delivered the baby and was not breastfeeding.

PPCM patients face an increased risk of thrombus formation, warranting consideration of anticoagulants, particularly for those with severe left ventricular dysfunction. Bromocriptine has been explored as a prolactin blocker in PPCM cases, aiming to inhibit the enhanced oxidative stress-mediated cleavage of nursing hormone prolactin into a 16-kDa form that is antiangiogenic and proapoptotic. This intervention is attempted to prevent the development of PPCM. 

The reduction of pulmonary edema by furosemide is attributed to its pleiotropic effects. By blocking the sodium–potassium 2 chloride co-transporter in the ascending loop of Henle, furosemide enhances urinary output, thereby reducing positive cumulative fluid balance. Additionally, furosemide quickens the intravascular refill time, facilitating the resolution of interstitial edema. This, in turn, lowers interstitial fluid pressure, leading to a decrease in mechanical compression on systemic capillary and systemic vein pressure. Consequently, MCFP and CVP are reduced. The decreased CVP, in turn, aids in increasing venous return by minimizing the pressure gradient between MCFP and CVP. This contributes to an enhanced cardiac output, ultimately leading to improved tissue oxygenation. Starling’s law is the most common theory used right now to describe hemodynamic cardiovascular instability, Guyton’s perspective is yet to be used commonly in this matter. Further research is needed to understand more about the mechanism of how furosemide allows interstitial fluid to shift to intravascular space.

## Conclusion

A relationship exists between a negative cumulative fluid balance and a decrease in central venous pressure following early furosemide therapy. It's crucial not to diminish suspicions of PPCM when preeclampsia is present, as doing so may delay appropriate treatment and potentially elevate mortality rates.

## Data Availability

All medical data is available on the paper. Personal data of the patient is unavailable due to privacy.

## Abbreviations

ASA: American Society of Anesthesiologists
CPAP: Continuous positive airway pressure
CO: Cardiac output
CVP: Central venous pressure
ICU: Intensive care unit
MCFP: Mean capillary filling pressure
PPCM: Peripartum cardiomyopathy
RAP: Right atrium pressure
SGPT: Serum pyruvic-oxaloacetic transaminase
SGOT: Serum glutamic-oxaloacetic transaminase
VR: Venous return
